# Effects of small extracellular vesicles isolated from pleural effusion on lung cancer cell proliferation and migration

**DOI:** 10.1007/s13577-025-01322-8

**Published:** 2025-11-15

**Authors:** G. Cammarata, A. Masucci, I. Giusti, V. Dolo, C. Di Sano, S. Taverna, E. Pace

**Affiliations:** 1 Institute of Translational Pharmacology (IFT), National Research Council (CNR), 90146 Palermo, Italy; 2 Department of Biomedicine, Neurosciences and Advanced Diagnostics, Institute of Clinical Biochemistry, Clinical Molecular Medicine, and Clinical Laboratory Medicine, University of Palermo, 90127 Palermo, Italy; 3 Department of Life, Health and Environmental Sciences, University of L’Aquila, L’Aquila, Italy

**Keywords:** Extracellular vesicles, Non-Small cell lung cancer, Congestive heart failure, Pleural effusion, MiR-21-5p

## Abstract

Pleural effusion (PE) is a common clinical manifestation associated with advanced stages of both malignant and non-malignant diseases. PE frequently occurs in advanced non-small cell lung cancer (NSCLC) and contributes to tumor progression. NSCLC accounts for more than 85% of the lung cancers and remains a problem worldwide due to its late diagnosis and low rate of response to treatment. Extracellular vesicles (EVs) present in PE are emerging as key mediators of intercellular communication, capable of transferring oncogenic signals through their molecular cargo. Among these molecules, microRNAs (miRNAs) are increasingly recognized as important drivers of cancer progression. miR-21 is a representative onco-miRNA, involved in lung cancer progression; moreover EV‐miR‐21 upregulation at the pre‐dissemination stage promotes cancer cell survival in the pleural cavity. This study compares, for the first time, the functional role of EVs isolated from malignant PE in NSCLC patients (NSCLC-PE-EVs) with those isolated from PE in patients with congestive heart failure (CHF-PE-EVs), focusing on their ability to modulate lung cancer cell behavior. The effects of these EVs were evaluated on COLO699 lung adenocarcinoma cells with proliferation, migration, and gene expression assays. NSCLC-PE was found to contain approximately twice the amount of EVs compared to CHF-PE. NSCLC-PE-EVs were enriched in the oncogenic miR-21-5p, while CHF-PE-EVs had higher levels of the tumor-suppressive miR-126-3p. Only NSCLC-PE-EVs induced dose-dependent increases in COLO699 cell proliferation and migration, consistent with elevated miR-21-5p expression. Functional studies confirmed that miR-21-5p mediates these effects by downregulating PTEN and PDCD4, and by upregulating MMP9 expression. Our findings show that NSCLC-PE-EVs promote malignant phenotypes in lung cancer cells via the transfer of miR-21-5p.

**Supplementary Information:**

The online version contains supplementary material available at 10.1007/s13577-025-01322-8.

## Introduction

Lung cancer (LC) remains the leading cause of cancer-related incidence and mortality worldwide, with approximately 2.2 million new cases and 1.8 million deaths reported annually [1–4]. Among its subtypes, non-small-cell lung cancer (NSCLC) accounts for over 85% of cases [5]. Pleural effusion (PE) is a frequent clinical manifestation associated with advanced stages of both malignant and non-malignant diseases and is typically indicative of poor prognosis [6]. PE originates from the lung interstitium and pleural capillaries and contains various cell types, including macrophages, lymphocytes, mesothelial cells, neutrophils, and eosinophils [7]. In malignant pleural effusion (MPE), tumor cells disrupt lymphatic drainage and increase vascular permeability, leading to the accumulation of fluid between the visceral and parietal pleura [8]. PE occurs in 60–70% of cancer patients and is also common in non-malignant conditions [9]. It affects approximately 30–40% of individuals with cardiovascular diseases, particularly congestive heart failure (CHF), which represents the most common non-malignant cause of PE. In CHF, pleural effusion typically results from elevated hydrostatic pressure in the pulmonary circulation, leading to fluid transudation into the pleural space. Unlike MPE, these effusions are usually bilateral, transudative, and respond well to appropriate diuretic therapy. Among cancer patients, MPE is observed in approximately 30% of those with advanced NSCLC, followed by individuals with breast cancer and lymphoma [10]. MPE constitutes a complex tumor microenvironment (TME) composed of detached tumor cells, fibroblasts, immune cells, and extracellular matrix components [11, 12]. Compared to plasma, MPE is particularly enriched in tumor-derived components such as circulating tumor cells (CTCs), circulating tumor DNA (ctDNA), cell-free RNA (cfRNA), tumor-educated cells (platelets and macrophages), and non-coding RNAs (ncRNAs) [13]. Within this environment, extracellular vesicles (EVs) and soluble factors actively contribute to tumor progression. EVs are a heterogeneous population of nano-sized, double-membrane vesicles released by various cell types under both physiological and pathological conditions [14], and are classified based on their size, biogenesis, and release mechanisms [15–18]. EVs carry diverse bioactive cargoes, including proteins, lipids, and nucleic acids [19–22], and have emerged as promising biomarkers for monitoring tumor progression and evaluating treatment response [23, 24]. EVs can promote cancer cell proliferation [25], inhibit anti-tumor immune responses [26], and facilitate intercellular communication within the TME [27]. Among the molecular cargoes of EVs, microRNAs (miRNAs) play a central role in regulating gene expression and influencing cancer behavior. These small ncRNAs (19–22 nucleotides) bind to complementary sequences in the 3′ untranslated regions (3′UTRs) of target mRNAs, leading to mRNA degradation or translational repression [28]. Several EV-derived miRNAs (EV-miRNAs) have been investigated for their potential as biomarkers in tumor subtype classification, early diagnosis, prediction of therapeutic response, and prognosis in NSCLC [29, 30]. Of particular interest are miR-21-5p and miR-126-3p, which have been implicated in various malignancies, including NSCLC, colorectal cancer, and renal cell carcinoma (RCC).

Recent studies have shown that PE-derived EV-miRNAs may serve as promising non-invasive biomarkers for NSCLC diagnosis and prognosis. For instance, miR-21, miR-200 family members, and miR-182 have been consistently reported at elevated levels in EVs from MPE of NSCLC patients compared to benign conditions, highlighting their potential diagnostic value [31–33]. Furthermore, EV-miRNA profiling from PE has been explored as a tool to predict therapeutic response and tumor aggressiveness. For example, miR-375 and miR-203a-3p have been associated with lung cancer progression and could help stratify patients for treatment [34, 35] The miR-21-5p is one of the most consistently upregulated onco-miRNAs in NSCLC and is associated with poor clinical outcomes. This miRNA exerts its biological effects primarily through the post-transcriptional repression of tumor suppressor genes[36]. It functions as an oncogene by targeting tumor suppressors such as phosphatase and tensin homolog (PTEN) and programmed cell death 4 (PDCD4) [37, 38]. Loss of PTEN expression, frequently observed in NSCLC, may result from both epigenetic and post-transcriptional mechanisms [39]. The miR-21-5p directly targets PDCD4, a tumor suppressor involved in apoptosis and inhibition of invasion. Downregulation of PDCD4 by miR-21 contributes to enhanced tumor cell migration, invasion, and metastasis [40]. Although MMP9 is not a validated direct target of miR-21, its expression is frequently upregulated downstream of miR-21 activity. This miRNA exerts its regulatory effects through the coordinated repression of multiple tumor suppressor genes involved in converging signalling pathways [41]. Specifically, the downregulation of PTEN and PDCD4 by miR-21 leads to the activation of oncogenic cascades, including the PI3K/AKT and NF-κB pathways, both of which are known to transcriptionally induce MMP9 expression. MMP9, a matrix metalloproteinase implicated in extracellular matrix remodelling, plays a pivotal role in promoting tumor invasion and metastatic dissemination [42, 43]. The selective packaging of miRNAs into EVs, including miR-21[37], is a regulated process influenced by cellular stress, oncogenic signalling, and hypoxia, conditions common in TME[44]. Specifically, miR-21 upregulation in tumor cells can result from aberrant activation of pathways such as STAT3 and NF-κB [42], which also facilitate its incorporation into EVs via RNA-binding proteins like hnRNPA2B1 or SYNCRIP [45, 46]. Recently miR-21 has been found to be aberrantly expressed in MPE [47]. In NSCLC-PE, EVs enriched in miR-21 may represent an adaptive mechanism by which tumor cells facilitate progression, immune evasion, and metastatic dissemination [48].

EV-associated miR-21 can induce macrophage polarization toward an M2 phenotype, characterized by immunosuppressive activity and diminished anti-tumor responses [49]. In turn, EVs released by M2 macrophages contribute to reduced tumor immunogenicity and promote resistance to immunotherapy [50]. EVs isolated from MPE have also been shown to contain miR-21 [51]. Additionally, miRNAs carried by PE-derived EVs have demonstrated potential in distinguishing between benign and malignant PEs [52]. These findings support the use of PE as a valuable source for investigating EV-miRNAs in NSCLC, within a liquid biopsy approach.

In contrast, miR-126-3p acts as a tumor suppressor and is frequently downregulated in various malignancies, including leukemia and gastric cancer. It has been shown to inhibit cancer progression by downregulating vascular endothelial growth factor (VEGF) expression [53, 54]. In NSCLC, reduced levels of EV-associated miR-126-3p have been linked to advanced disease stages, and its restoration impairs tumor cell proliferation, migration, and metastatic potential by targeting chemokine receptor 1 (CCR1) [53, 55]. This study investigates the role of EVs isolated from the pleural effusion of NSCLC patients in tumor progression. We evaluate the diagnostic and functional relevance of miR-21-5p within PE-derived EVs as potential biomarkers of MPE. Due to the lack of specific markers for the definitive classification of EV subpopulations, we refer here to small extracellular vesicles (sEVs) as those measuring less than 200 nm in diameter [56, 57]. In this study, we present for the first time a comparative analysis of sEVs from NSCLC- and CHF-derived PEs, revealing their differential effects on LC cell proliferation and migration. These findings highlight the pivotal role of NSCLC-derived PE-sEVs in modulating TME interactions and suggest their potential utility as both functional mediators and diagnostic biomarkers in LC progression.

## Materials and methods

### Patient inclusion

PEs were collected by therapeutic thoracentesis from patients with non-small-cell lung cancer (NSCLC) and Congestive Heart Failure (CHF) (n = 5 for each group, age range 50–78 years) from 2009 to 2010 at Hospital V. Cervello, Palermo Italy. Hospital V. Cervello provided IBIM-CNR with pleural effusions in the context of an agreement between Hospital V. Cervello-IBIM-CNR (prot. IBIM-CNR 2472- 30.07.2009). Local Etic Committee (Etic Committee Palermo 1–05/2023) approved as retrospective study the use of collected pleural effusions for research purposes. The use of these samples for research purposes, in the absence of informed consent, complied with art. 110 of the current Privacy Legislation (last updated June 5, 2024). PEs were drawn into polypropylene bags containing heparin (10–20 U/ml) and were subsequently centrifuged at 400 *g* for 10 min. Cell-free fluids have been aliquoted and frozen at −80° C for the following experiments [12].

### Cell lines and reagents

Human lung adenocarcinoma cell line, derived from pleural effusion, COLO-699 cell line was obtained from ATCC (Manassas, VA, USA). COLO-699 cells were grown in RPMI-1640 (Euroclone, Pero, MI, Italy) supplemented with 10% Fetal Bovine Serum (FBS, Euroclone), 100 U/ml penicillin, 100 µg/ml streptomycin (Euroclone), 1% MEM Non-Essential Amino Acids Solution (Euroclone) and 2% Hepes (Euroclone).

### Extracellular vesicles isolation

EVs were isolated from 10 ml of PE collected from 10 patients, 5 with NSCLC and 5 with CHF; PE was filtered with 0.4 µm filter, EVs were isolated after centrifugation at 800×g for 5 min, 3000×g for 15 min, 10000×g for 30 min, 10000×g for 45 min, and 100,000×g ultracentrifugation for 1 h and 45 min at 4 °C. EVs pellet was washed and then resuspended in PBS (Euroclone). The concentration of EVs was determined by quantifying total protein content using the Bradford assay [58]. Briefly, 10 µl of EVs resuspended in PBS were added to 200 µl of Coomassie Protein Assay Reagent (Pierce, Rockford, IL, USA). The absorbance at 595 nm was measured with the spectrophotometer (SPECTROstar nano BMG LABtech, Ortenberg, Germany). The protein concentration was calculated using a standard curve of a dilution series of bovine serum albumin (BSA, Merck, Darmstadt, Germany) whose concentrations are known.

### Nanoparticle tracking analysis

Nanoparticle tracking analysis (NTA) was performed to analyze particle size distribution and concentration (particles/ml) using the NanoSight NS300 system (NanoSight Ltd., Amesbury, United Kingdom). EVs were resuspended in sterile, filtered PBS to generate a dilution in which 20–120 particles/frame were tracked; for each sample, 5 recordings of 60 s were performed (for a total of 1498 frames) and were captured and analyzed using NTA 3.1 software by applying optimized settings.

### Western blotting

EV-proteins were resolved by 10% sodium dodecyl sulphate-polyacrylamide gel electrophoresis (SDS-PAGE) in non-reducing conditions and with heating for CD63 or reducing conditions and with heating for CD9. After the electrophoresis, proteins were transferred to nitrocellulose membranes (GE Healthcare Life Sciences, Boston, United States of America) and non-specific binding sites were blocked by incubating membranes in 10% non-fat dry milk diluted in TBS-T (TBS plus 0.5% Tween-20), under agitation at RT for 90 min. Then, membranes were incubated overnight at 4 °C with the mouse monoclonal anti-CD63 (dilution 1:400; sc-59286) or the mouse monoclonal anti-CD9 (dilution 1:400; sc-13118) antibody; after being washed in TBS-T, the membranes were incubated with a goat anti-mouse IgG-HRP antibody (dilution 1:10,000; sc-2005) for 1 h. All antibodies were from Santa Cruz Biotechnology, Inc. (Dallas, Texas, United States of America) and were used after proper dilution in TBS-T containing 1% non-fat dry milk. After membrane washings in TBS-T, the reactive bands were detected and acquired as images with the documentation system on gel Alliance LD2 (UVItec, Cambridge, United Kingdom), using a chemiluminescence detection kit (Super Signal West Pico Chemiluminescent Substrate; Merck Life Sciences, Taufkirchen, Germany).

### Uptake of PE-EVs by COLO699 cells

PE-EVs were labelled with PKH26 (Merck, Darmstadt, Germany), according to the manufacturer’s instructions. Briefly, sEVs, collected by 100,000 × g ultracentrifugation, were incubated with PKH26 for 10 min at room temperature. Labelled EVs were washed in PBS, by ultracentrifugation and resuspended in a low serum medium. COLO699 cells were grown on coverslips coated with type I collagen (Calbiochem, Darmstadt, Germany) in 12-well plates at a density of 6 × 10^4^ cells per well. COLO-699 cells were incubated with 20 µg/ml of labelled sEVs for 1–3 h at 37°, 5% CO_2_ and, after that, stained with ActinGreen^™^ 488 Ready ProbesR Reagent (Life Technologies, Carlsbad, CA, USA) that binds F-actin with high affinity. Nuclei were stained with Hoechst (Molecular Probes, Life Technologies, Carlsbad, CA, USA) and analysed by confocal microscopy (AX R, Nikon, Amstelveen, Netherlands). The semi-quantitative analyses were performed by the software ImageJ.

### MTS assay

Cell proliferation of COLO-699 cell line was evaluated by Celltiter 96 Aqueous One Solution Cell Proliferation assay (PROMEGA, Madison WI USA) according to manufacturer’s instructions. COLO-699 cells have been treated with 1, 10, and 20 µg/ml of PE-EVs collected by 5 patients with NSCLC and 5 with CHF, PE deprived of sEVs the same volume from which 20 μg/ml of sEVs was isolated and PE. PE deprived of sEVs was obtained from PE after the ultracentrifugation to isolate the sEVs. Briefly, MTS [3-(4,5-dimethylthiazol-2-yl)−5-(3-carboxymethox-yphenyl)−2-(4-sulfopheyl)2H-tetrazolium] solution was added to each well and incubated for 20 min at 37 °C, 5% CO_2_. The absorbance was read at 490 nm by a microplate reader, SPECTROstar^®^ Nano (BMG LABTECH, Ortenberg, Germany).

### Motility assays

Motility assays were performed by Boyden chamber (NeuroProbe Inc., Cabin John, MD, USA). Briefly, COLO-699 cells (2 × 10^6^/ml), control and transfected with miR-21 mimic and inhibitor (Applied Biosystems, Foster City, CA, USA), were suspended in serum-free RPMI 1640 medium supplemented with 0.1% BSA plus an increased amount of PE-EVs (1–10–20 µg/ml), PE deprived of sEVs the same volume from which 20 μg/ml of sEVs was isolated and PE, in transwell chemotaxis above 8 µm pore filters, and exposed to RPMI 1640 with 10% FBS. Filters were removed after 18 h, fixed in methanol, and stained with Diff-Quick (Medion Diagnostics GmbH, Düdingen, Switzerland). Each condition was tested in three independent experiments; the number of migrating cells, in five high-power fields per well, was counted.

### RNA extraction

The total RNA was extracted by COLO699 cell line, treated or not with EVs, using RNAspin mini kit (GE Healthcare Science, Uppsala, Sweden), according to the manufacturer’s instruction. The RNA concentration was assessed using the SpectroStar Nano reader (BMG Labtech, Ortenberg, Germany). For this study, only RNA with a ratio of A260/280 from 1.9 to 2 has been used.

### TaqMan RT-qPCR for miR-126-3p, miR-21-5p, pre-miR-21-5p, PTEN, PDCD4 and MMP9

The cDNA was synthesized from the total RNA, using the iScript cDNA synthesis kit (Biorad, Hercules, CA, USA), according to the manufacturer’s protocol. For the reverse transcription of miR-21-5p (Assay ID: 000397, Applied Biosystems, Foster City, CA, USA) and miR-126-3p (Assay ID: 000450, Applied Biosystems, Foster City, CA, USA), 1 µg of total RNA was incubated with specific primers using the TaqMan^™^ microRNA assay and the TaqMan miRNA RT kit (Applied Biosystems, Foster City, CA, USA), according to the manufacturer’s protocol. Mature miR-21-5p and pre-miR-21-5p expression were evaluated using specific TaqMan microRNA assay ID: 000397, and ID: 4,331,182 respectively (Applied Biosystems, Foster City, CA, USA), with Real-time quantitative PCR (RT-qPCR) by Step One Plus real-time PCR system (Applied Biosystems, Foster City, CA, USA). MiR-21-5p expression in cells was normalized with RNU6 using the TaqMan^™^ microRNA assay (Assay ID: 001973, Applied Biosystems, Foster City. miR-21-5p expression in EVs was normalized with miRNA-30a using the TaqMan^™^ microRNA assay (Assay ID: 478,388, Applied Biosystems, Foster City) [59]. PTEN gene expression was evaluated with specific FAM-labelled probe and primers, as part of the TaqMan gene expression assay for PTEN (Hs02621230_s1 Applied Biosystems, Foster City, CA, USA), PDCD4 (Hs03677934_s1 Applied Biosystems, Foster City, CA, USA) and MMP9 (Hs00957562_m1 Applied Biosystems, Foster City, CA, USA) with RT-qPCR using a Step One Plus real-time PCR system (Applied Biosystems, Foster City, CA, USA). The gene expression was normalized to GAPDH with a Taq-Man gene expression assay for GAPDH (Hs03929097_g1, Applied Biosystems, Foster City, CA, USA) as a housekeeping gene. For the amplification, the reaction mixtures were incubated at 95 °C for 15 min, followed by 40 amplification cycles of 94 °C for 15 s, 55 °C for 30 s, and 70 °C for 30 s. Triplicate samples and inter-assay controls were used. For the normalization of RT-qPCR data, the 2-∆CT method has been used.

### Analysis of miR-21-5p and miR-126-3p in PE-EVs

Total RNA was extracted from sEVs isolated from PE of 5 patients with NSCLC and 5 with CHF. cDNA was synthesized from the extracted RNA, and RT-qPCR for miR-21-5p and miR-126-3p was performed as described above.

### COLO699 cells transfection with miR-21 mimic or inhibitor

The transfection with miR-21 inhibitor or mimic (Applied Biosystems, Foster City, CA, USA) was performed according to the manufacturer's instructions. COLO699 cells were seeded in a 12-well plate in 500 μl of Opti-MEM culture medium (Gibco^™^ Waltham, MA USA 02451). For the transfection COLO699 cells were at 80% confluent in RPMI without serum. miR-21 inhibitor or mimic (1 μM) was diluted in Opti-MEM. The cells were transfected using Lipofectamine 2000 Reagent (Life Technologies, Carlsbad, CA, USA) according to the manufacturer's instructions for 18 h. Transfection efficiency was evaluated by RT-qPCR for miR-21-5p.

### Statistical analysis

Comparisons between PE from NSCLC and CHF patients were analysed using the non-parametric Mann–Whitney test. Comparisons among multiple groups, for data with normal distribution and homogeneity of variance, were assessed by one-way analysis of variance (ANOVA). For each experimental condition (PE, PE-sEVs, and PE-sEV-depleted fractions) the biological samples were obtained from 5 NSCLC and 5 CHF patients. Data, for each patient sample, are expressed as mean ± standard deviation (SD) from three independent experiments.

For in vitro experiments, data from Colo699 cells are also expressed as mean ± SD from three independent experiments.

Differences were considered statistically significant at p ≤ 0.05 and highly significant at p ≤ 0.01. Statistical analyses were performed using GraphPad Prism 6 software.

## Results

### EV isolation, characterization and mir-21-5p EV content quantification

**Fig. 1:**
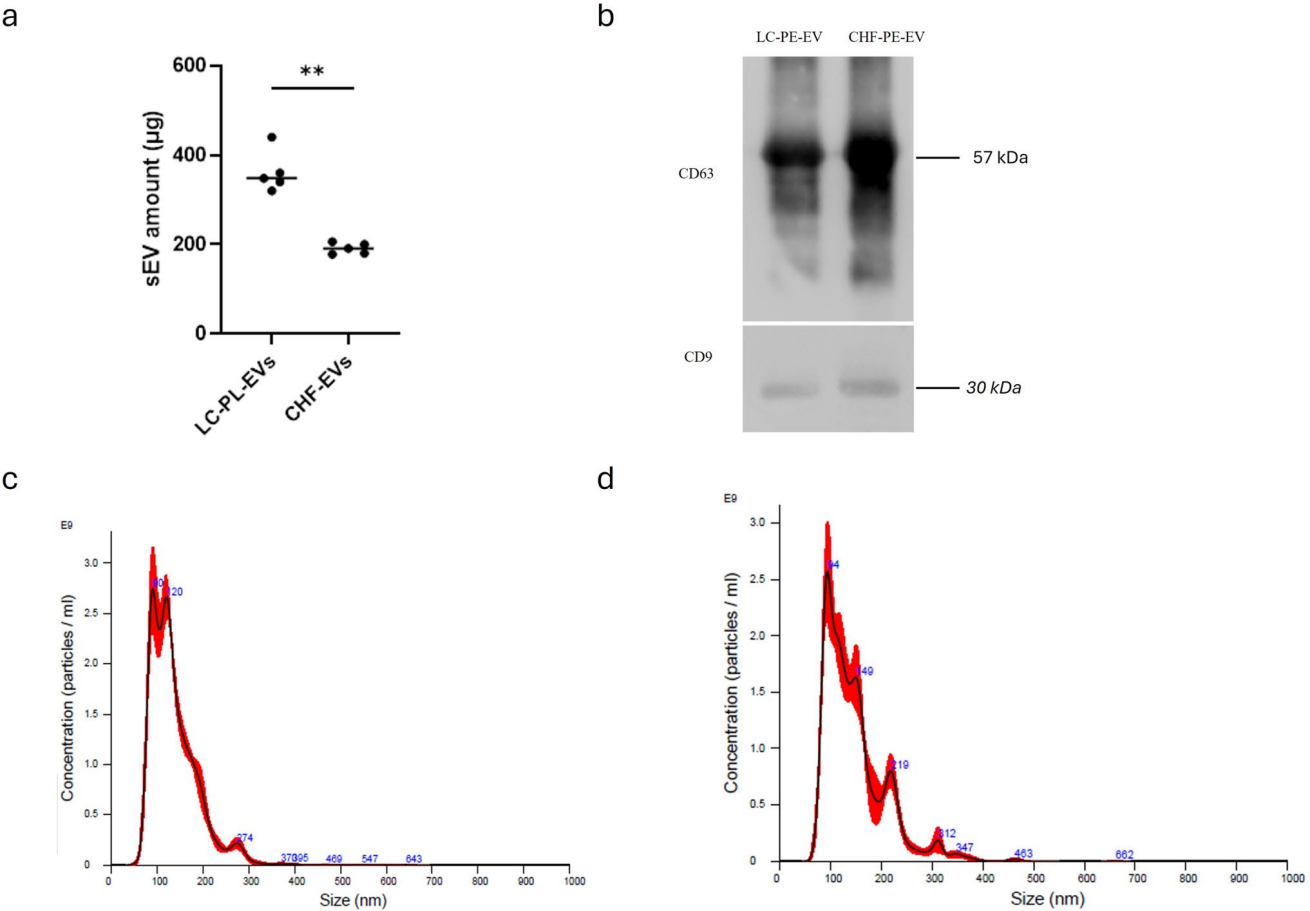
EV-characterization: Amount of sEVs (µg of EV-protein content) isolated from 10 ml PE of patients (five patients per group) with NSCLC and CHF (**a**). Values are the mean ± SD of three independent experiments, for each patient **p ≤ 0.01. In all figures NSCLC is indicated as LC for simplicity. Detection by western blotting of CD63 and CD9 in LC-PE-EVs and CHF-PE-EVs (**b**). NTA of EVs isolated from LC-PE (**c**) and CHF-PE (**d**)

**Fig. 2:**
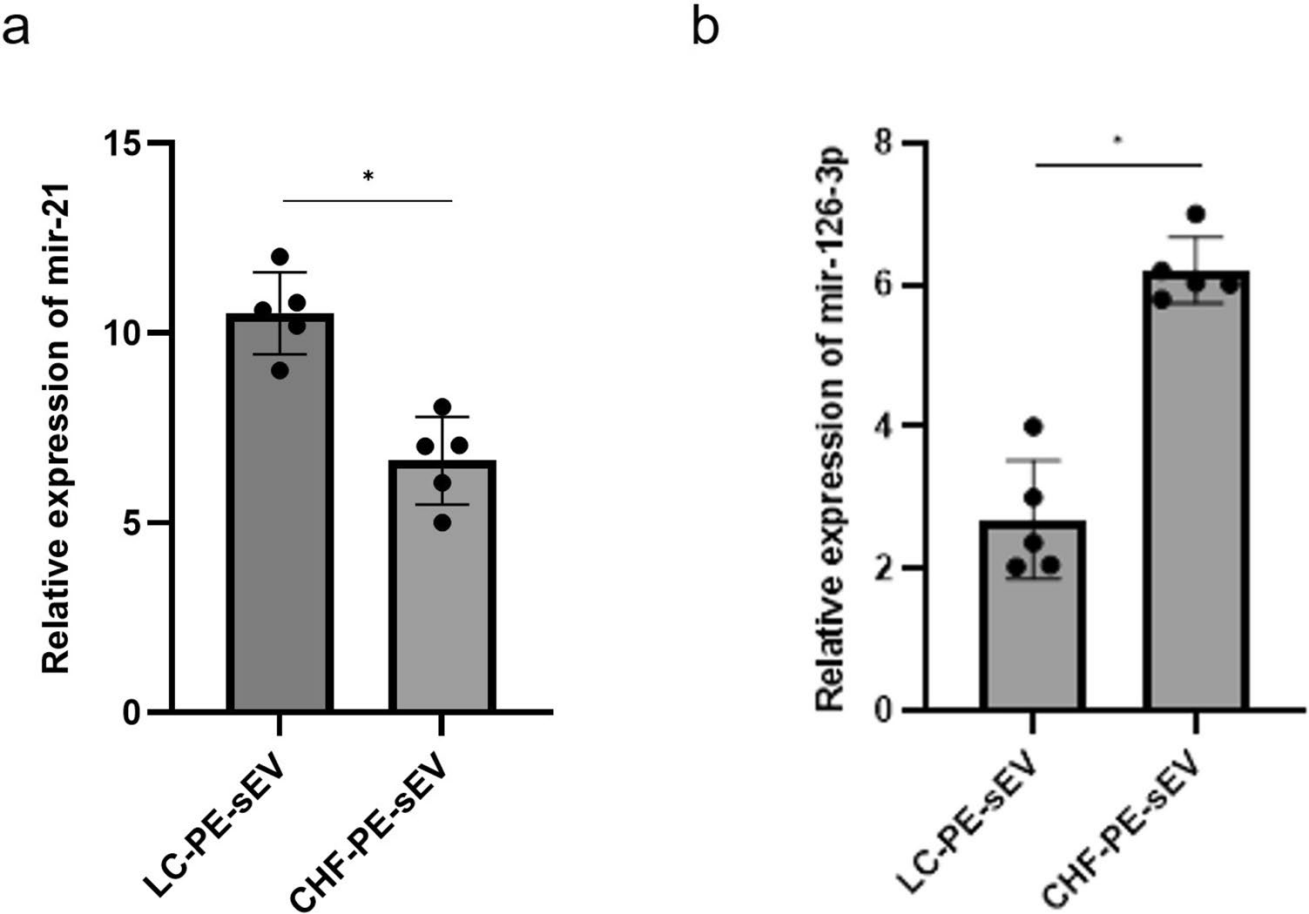
miRNA content in PE-sEVs: Relative expression levels of miR-21-5p (**a**) and miR-126-3p (**b**) were measured by RT-qPCR in NSCLC-PE-sEVs and CHF-PE-sEVs. Data are presented as fold change. Results are based on samples from 10 different patients (5 NSCLC and 5 CHF). Values are the mean ± SD in three independent experiments *p ≤ 0.05

EVs were isolated from 10 ml PE collected from patients with NSCLC and CHF (5 patients per group) to compare malignant and benign PE, by an optimized protocol based on differential centrifugation and ultracentrifugation. The concentration of EVs was determined by quantifying total protein content using the Bradford assay. NSCLC-PE contained a higher amount of EVs (361 ± 46.18 µg) than CHF-PE, (190 ± 12.21 µg), (Fig. 1 a). EVs were analysed by Western blotting using antibodies specific for the well-known EV markers CD63 and CD9 [56] (Fig. 1b). Nanoparticle tracking analysis (NTA) indicated that particles isolated from NSCLC-PE and CHF-PE have a similar diameter with a mean of 136.6 ± 0.7 nm and 146.5 ± 1.0 nm respectively (Fig. 1 c, d). Considering the particles' diameter size, here we refer to this EV population as small extracellular vesicles (sEVs). In PE-EVs have also been evaluated the presence of miR-21-5p and miR-126-3p, that have been found both in NSCLC-PE-sEVs and CHF-PE-sEVs isolated from patients with NSCLC and CHF. As demonstrated by RT-qPCR assays, miR-21-5p (Fig. 2a) was more abundant in NSCLC-PE-sEVs, conversely miR-126-3p was more abundant in CHF-PE-sEVs (Fig. 2b).

### sEVs internalization from COLO699 cells

**Fig. 3:**
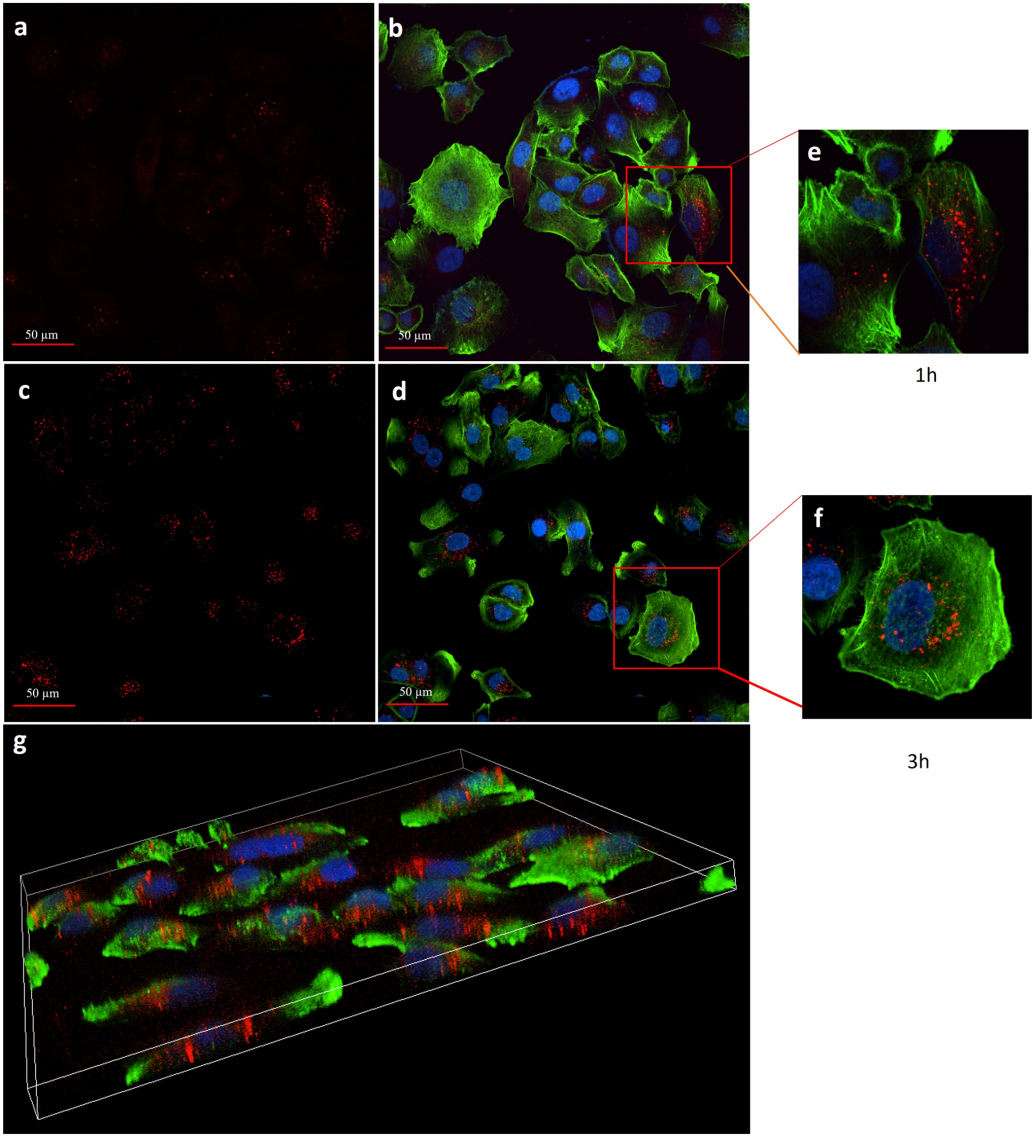
COLO699 cells internalize NSCLC-PE-sEVs: Confocal microscopy analysis of COLO699 cells treated, for 1 and 3 h, with 20 μg/ml of NSCLC-PE-sEVs. COLO699 cells were stained with ActinGreen (green), nuclear counterstaining was performed using Hoescht (blue); NSCLC-PE-sEVs were labelled with PKH26 (red). Red channel (**a**, **c**), merge images (**b**, **d**). Detail of merge figure (**e**, **f**). Confocal-imaging-based 3D reconstruction of COLO699 cells treated with with 20 μg/ml of LC-PE-sEVs (**g**). Magnification (60×). Scale bar 50 µm

The first step to demonstrate the involvement of PE-EVs in cancer progression has been to evaluate their tropism to NSCLC cells, using as target the adenocarcinoma cell line derived from pleural effusion, COLO699. The ability of PE-sEVs, to be internalized by COLO699 cells, was investigated by confocal microscopy analysis. The uptake of isolated NSCLC-PE-sEVs and CHF-PE-sEVs (20 μg/ml) labelled with PKH-26 by COLO699 cells, at 1 and 3 h, has been analysed. COLO699 cells internalized NSCLC-PE-sEVs (Fig. 3 a–g) and CHF-PE-sEVs (Supplemental Fig. 1a–f), in a time-dependent manner, as indicated by semi-quantitative analysis (Supplemental Fig. 2a and b).

### *Effects of PE and PE-EVs from *NSCLC* and CHF patients on COLO699 cell proliferation*

**Fig. 4:**
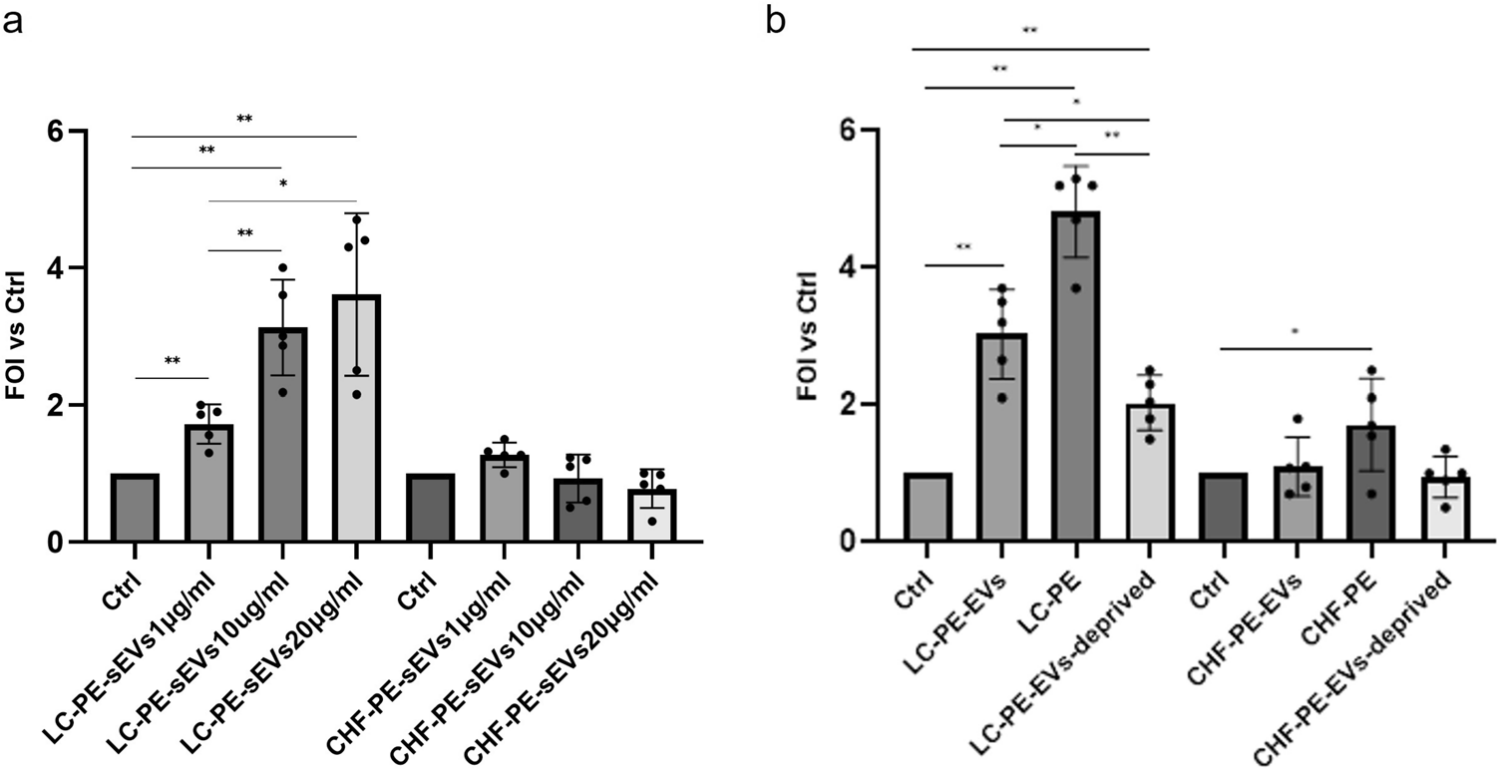
COLO699 cell proliferation: **a** COLO699 cells were treated with increasing concentrations (1, 10, and 20 μg/ml) of NSCLC-PE-sEVs and CHF-PE-sEVs **b** COLO699 cells were treated with 20 μg/ml of NSCLC-PE-sEVs, NSCLC-PE, NSCLC-PE-sEV-deprived, CHF-PE-sEVs, CHF-PE, and CHF-PE-sEV- deprived. Cell proliferation is expressed as fold of induction (FOI) relative to untreated control cells (Ctrl). Results are based on samples from 10 different patients (5 NSCLC and 5 CHF) Values are the mean ± SD in three independent experiments *p ≤ 0.05, **p ≤ 0.01

To investigate the role of NSCLC-PE-sEVs in cell proliferation, COLO699 cells were treated for 18 h with different concentrations (1, 10, and 20 μg/ml) of NSCLC-PE-sEVs and CHF-PE-sEVs,. The results indicated that NSCLC-PE-sEVs induced COLO699 cell proliferation in a dose-dependent manner. In contrast, CHF-PE-sEVs had no statistically significant effect on COLO699 cell proliferation (Fig. 4a). These results demonstrate that NSCLC-PE effect on cell proliferation depends largely on the presence of sEVs. Since NSCLC-PE contains a large amount of different active molecules, to demonstrate the role of PE-EVs, COLO699 cells were treated with 20 μg/ml of NSCLC-PE-sEVs, NSCLC-PE, and NSCLC-PE deprived of EVs, for 18 h.

The results indicated that all treatments positively stimulated COLO699 cell proliferation, then NSCLC-PE and NSCLC-PE-sEVs increase the proliferation more than NSCLC-PE deprived of EVs (Fig. 4b). Moreover, COLO699 cells were treated with 20 μg/ml CHF-PE-sEVs, CHF-PE and CHF-PE deprived of EVs, for 18 h. As shown in Fig. 4b, these treatments had less effect on COLO699 cell proliferation compared to MPE. Notably, treatment with CHF-PE-sEV-deprived showed no statistically significant effect on COLO699 cell proliferation.

### Effects of PE and -PE-EVs from NSCLC and CHF patients on COLO699 cell motility

**Fig. 5:**
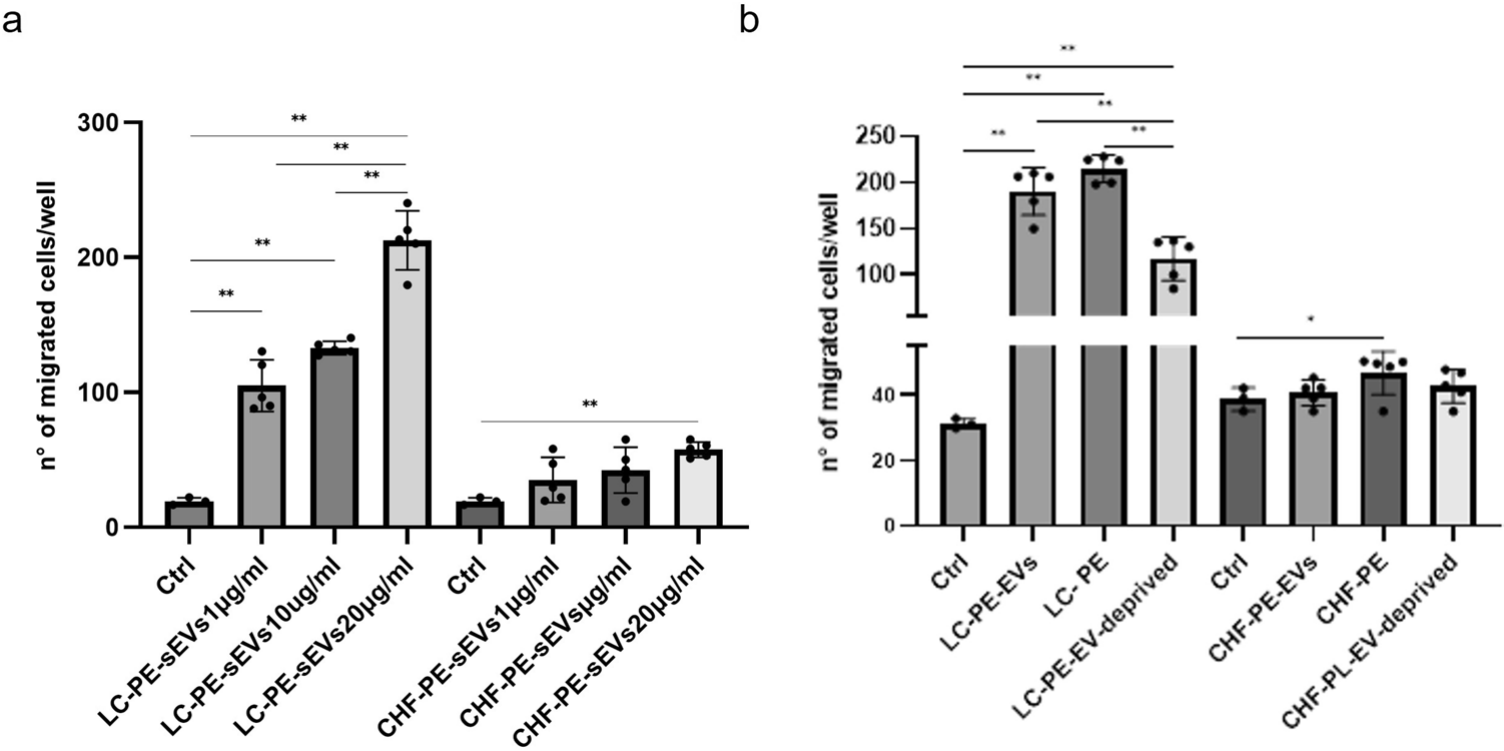
COLO699 cell migration: COLO699 cells treated with 1, 10, 20 μg/ml of NSCLC-PE-sEVs and CHF-PE-sEVs (**a**). COLO699 cells treated with NSCLC-PE-sEVs (20 μg/ml), NSCLC-PE, NSCLC-PE-sEV-deprived, CHF-PE-sEVs (20 μg/ml), CHF-PE, and CHF-PE-sEV-deprived. Results are based on samples from 10 different patients (5 NSCLC and 5 CHF). Values are the mean ± SD in three independent experiments *p ≤ 0.05, **p ≤ 0.01

Cell migration is a crucial step for many biologic processes including cancer progression [60]; to investigate the role of NSCLC-PE-sEVs on NSCLC cell migration, COLO699 cells were treated with different concentrations (1–10–20 μg/ml) of NSCLC-PE-sEVs and CHF-PE-sEVs, for 18 h. The results indicated that NSCLC-PE-sEVs induced COLO699 cell migration in a dose-dependent manner. Treatment with CHF-PE-sEVs had a less marked effect on COLO699 cell migration compared to NSCLC-PE-sEVs (Fig. 5a). Furthermore, to investigate NSCLC-PE effects, COLO699 cells were treated with 20 μg/ml of NSCLC-PE-sEVs, NSCLC-PE and NSCLC-PE deprived of sEVs. The results indicated that NSCLC-PE and NSCLC-PE-EVs induced COLO699 cell migration more than NSCLC-PE deprived of EVs (Fig. 5b). COLO699 cells were treated with 20 μg/ml CHF-PE-sEVs, CHF-PE and CHF-PE deprived of EVs, for 18 h. As shown in Fig. 5b, the treatments with CHF-PE-sEVs and CHF-PE had a less marked effect on COLO699 cell migration compared to NSCLC-PE-sEVs and NSCLC-PE-sEVs. The treatment with CHF-PE-sEV-deprived showed no statistically significant effect on COLO699 cell migration. These results indicate that sEVs in NSCLC-PE have also a key role in cell motility. Representative images of COLO699 cell migration are shown in Supplemental Fig. 3.

### NSCLC-PE-EVs effect on miR-21-5p expression in COLO-699 cells

**Fig. 6:**
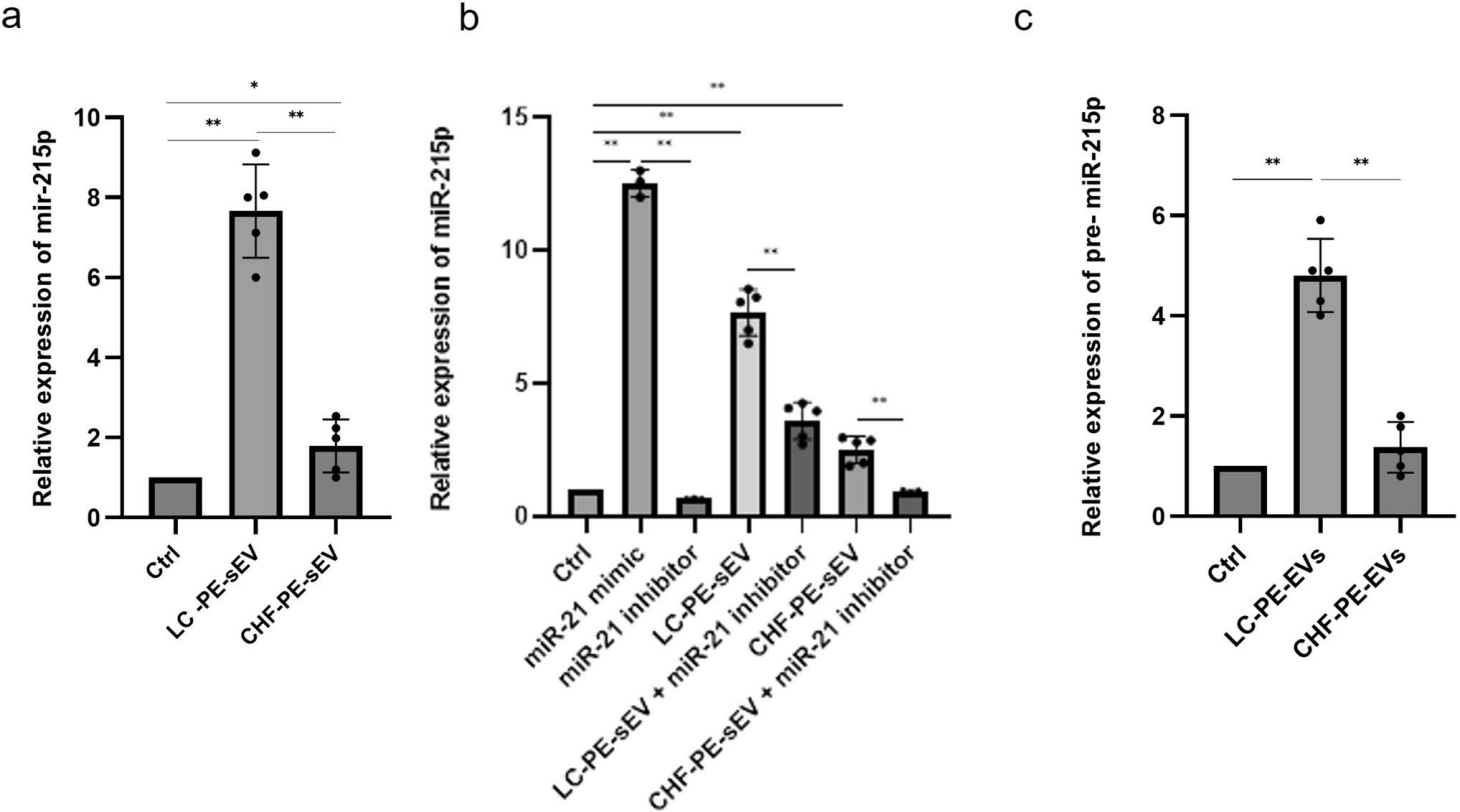
NSCLC-PE-sEV effect on COLO-699 cells miR-21-5p: NSCLC-PE-sEV effect on COLO-699 cells miR-21-5p: miR-21-5p expression in COLO699 cells treated with NSCLC-PE-sEVs and CHF-PE-sEVs (**a**). miR-21-5p expression in COLO699 cells transfected with miR-21-5p mimic and inhibitor and treated with NSCLC -PE-sEVs and CHF-PE-sEVs (**b**). Pre-miR-21-5p expression in COLO699 cells treated with NSCLC -PE-sEVs and CHF-PE-sEVs (**c**). The relative expression of miR-21-5p and pre-miR-21-5p is shown as fold change versus control. Results are based on samples from 10 different patients (5 NSCLC and 5 CHF) Values are the mean ± SD in three independent experiments *p ≤ 0.05, **p ≤ 0.01

Among the two types of sEVs, only NSCLC-PE-sEVs have a significant impact on cancer cell proliferation and motility. Since these sEVs are enriched in miR-21-5p (Fig. 2a), gain- and loss-of-function studies were conducted to investigate the role of this miRNA, which is present in PE. We investigated whether the effects of NSCLC-PE-sEVs on COLO-699 cells are mediated by miR-21-5p, and whether these vesicles can deliver miR-21-5p and/or induce its endogenous expression. To assess whether PE-sEVs modulate miR-21-5p levels in COLO-699 cells, we treated the cells with 20 μg/ml of sEVs isolated from NSCLC-PE and CHF-PE and analyzed miR-21-5p expression. As shown in Fig. 6a, miR-21-5p levels increased in COLO-699 cells treated with sEVs compared to untreated controls, and NSCLC-PE-sEVs induced a greater increase than CHF-PE-sEVs. To further confirm the involvement of miR-21-5p, we transfected COLO-699 cells with a miR-21 mimic or inhibitor. Transfection with the mimic resulted in elevated miR-21-5p levels. In cells treated with NSCLC-PE-sEVs and transfected with miR-21 inhibitor, the increase in miR-21-5p levels, induced by these sEVs, was reversed, whereas CHF-PE-sEVs had a less pronounced effect (Fig. 6b). To evaluate whether NSCLC-PE-sEVs also modulate endogenous miR-21-5p expression, we quantified the levels of its precursor, pre-miR-21, by RT-qPCR. As shown in Fig. 6c, treatment with 20 μg/ml of NSCLC-PE-sEVs led to an increase in pre-miR-21 expression, while no such effect was observed with CHF-PE-sEVs. These results indicate that NSCLC-PE-sEVs not only shuttle mature miR-21-5p into COLO-699 cells but also promotes its endogenous expression.

### Effects of miR-21-5p on COLO699 cell proliferation

**Fig. 7:**
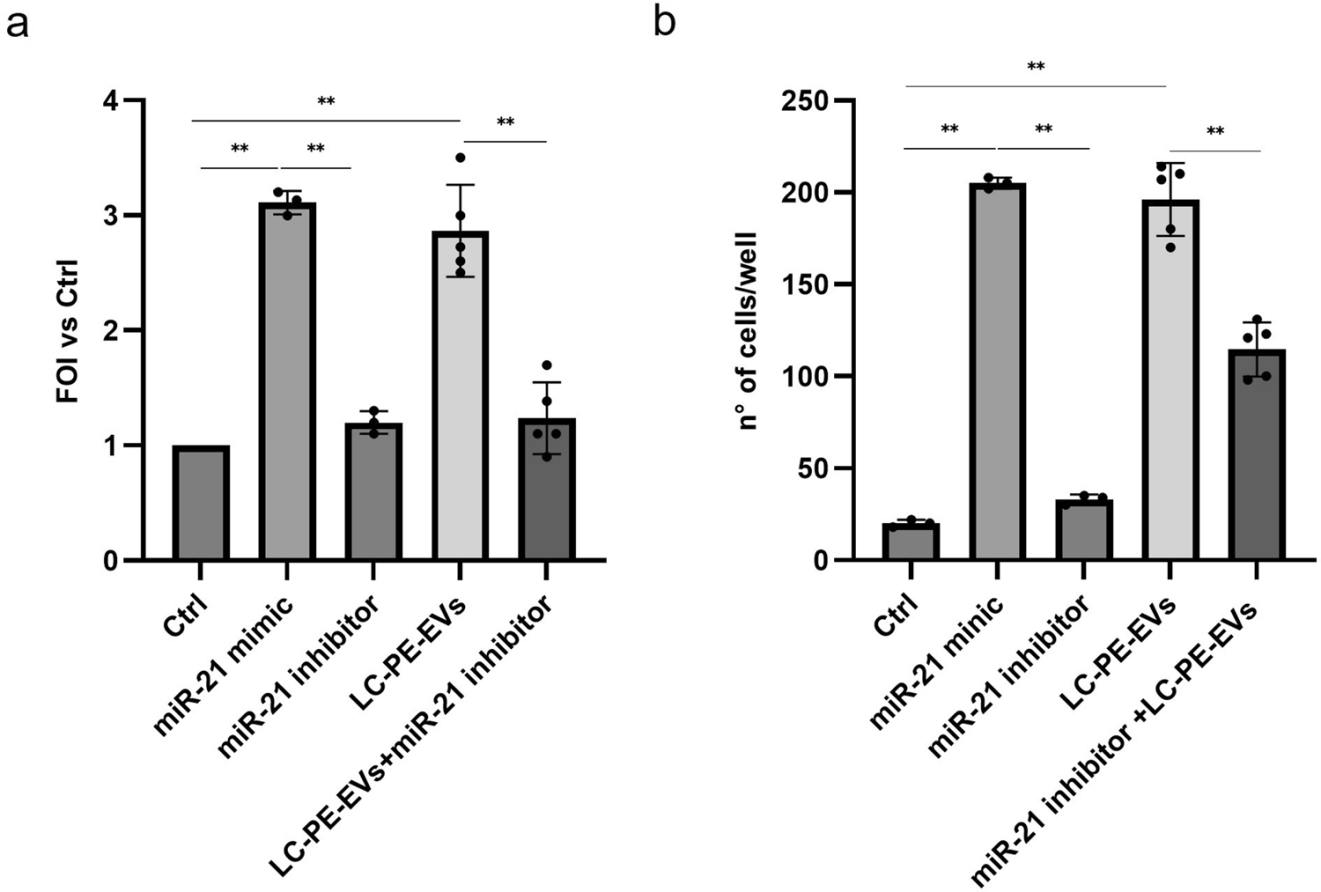
miR-21-5p delivered by NSCLC-PE-sEVs modulates COLO699 cell proliferation and migration: miR-21-5p delivered by NSCLC-PE-sEVs modulates COLO699 cell proliferation and migration: Proliferation of COLO699 cells transfected with miR-21-5p mimic and inhibitor and treated with 20 μg/ml of NSCLC-PE-sEVs (**a**). Migration of COLO699 cells transfected with miR-21-5p mimic and inhibitor and treated with NSCLC-PE-sEVs (**b**). The cell proliferation was expressed as fold of induction (FOI) versus untreated cells (Ctrl). Results are based on samples from 10 different patients (5 NSCLC and 5 CHF) and are presented as mean ± SD. *p ≤ 0.05, **p ≤ 0.01

Given that NSCLC-PE-sEVs were shown to promote COLO699 cell proliferation, we further investigated the role of miR-21-5p in this process by assessing the proliferation of COLO699 cells transfected with either a miR-21-5p mimic or inhibitor. Transfection with the miR-21-5p mimic promoted cell proliferation, whereas transfection with the miR-21-5p inhibitor resulted in a slight, but not statistically significant, reduction in proliferation. The transfection with the miR-21-5p inhibitor attenuated the proliferative effect induced by treatment with 20 μg/ml NSCLC-PE-sEVs (Fig. 7a). These results indicate that the increase in COLO699 cell proliferation mediated by NSCLC-PE-sEVs is, in part, dependent on miR-21-5p.

### Effects of miR-21-5p on COLO699 cell motility

As described above, NSCLC-PE-sEVs enhanced COLO699 cell motility. To further investigate the role of miR-21-5p in this process, we examined the migration of COLO699 cells transfected with either a miR-21-5p mimic or inhibitor. Transfection with the miR-21-5p mimic enhanced cell migration, whereas transfection with the miR-21-5p inhibitor had no effect on cell migration. Interestingly, transfection with the miR-21-5p inhibitor also attenuated the migration induced by treatment with 20 μg/ml NSCLC-PE-sEVs (Fig. 7b). These findings suggest that the increase in COLO699 cell migration mediated by NSCLC-PE-sEVs is, at least in part, dependent on miR-21-5p.

### NSCLC*-PE-EVs modulate PTEN PDCD4 and MMP9 gene expression in COLO699 cells*

**Fig. 8:**
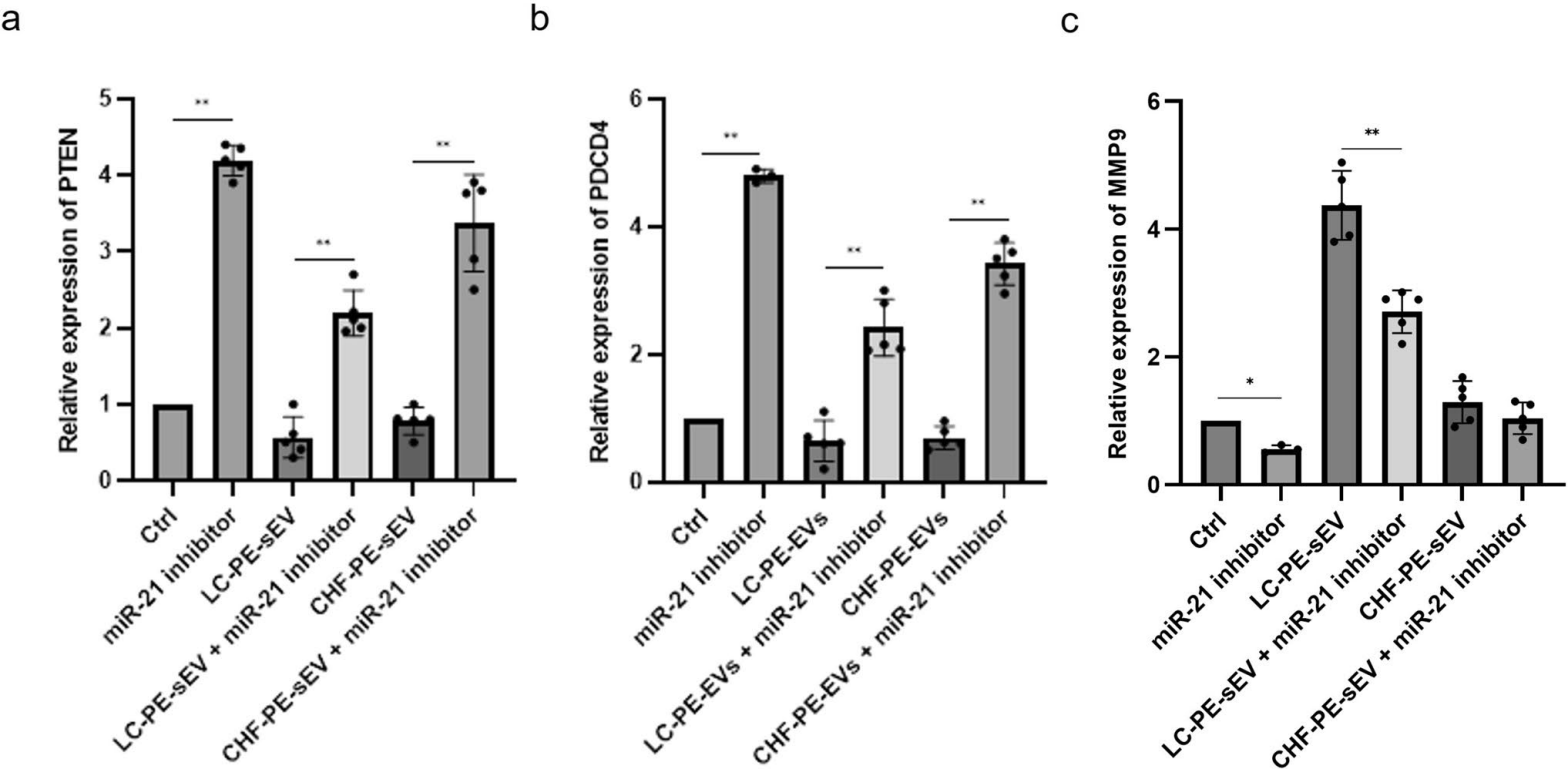
PTEN, PDCD4 and MMP-9 expression: PTEN, PDCD4 and MMP-9 expression: PTEN expression in COLO699 cells transfected with miR-21-5p inhibitor and treated with 20 μg/ml of NSCLC-PE-sEVs and CHF-PE-sEVs (**a**). PDCD4 expression in COLO699 cells transfected with miR-21-5p inhibitor and treated with NSCLC-PE-sEVs and CHF-PE-sEVs (**b**). MMP-9 expression in COLO699 cells transfected with miR-21-5p inhibitor and treated with NSCLC-PE-sEVs and CHF-PE-sEVs (**c**). The relative expression of PTEN, PDCD4 and MMP9 was showed as fold change versus control. Results are based on samples from 10 different patients (5 NSCLC and 5 CHF). Values are the mean ± SD in three independent experiments *p ≤ 0.05, **p ≤ 0.01

To investigate the molecular mechanisms by which miR-21-5p contributes to the effects mediated by NSCLC-PE-sEVs, we analyzed the expression of well-known miR-21-5p target genes in COLO699 cells. Transfection with a miR-21-5p inhibitor led to increased expression of PTEN and PDCD4, with a subsequent decrease of MMP9 expression. (Figs. 8a–c). In cells transfected with the miR-21-5p inhibitor, treatment with 20 μg/ml NSCLC-PE-sEVs reversed these effects, resulting in downregulation of PTEN (Fig. 8a) and PDCD4 (Fig. 8b), and upregulation of MMP9 (Fig. 8c). Treatment with 20 μg/ml CHF-PE-sEVs in miR-21-5p-inhibited cells similarly reversed the expression of PTEN and PDCD4 but had no statistically significant effect on MMP9 expression. These findings further support the role of NSCLC-PE-sEV-associated miR-21-5p in modulating key molecular targets involved in NSCLC progression.

## Discussion

The tumor microenvironment serves as a dynamic regulator of lung cancer progression and metastasis; within this context, EVs play a key role in facilitating cell-to-cell communication, transferring signals between cancer cells and normal cells, both within lung tissue and pleural effusion [61, 62]. EVs are involved in different steps of tumour progression and horizontal transfer of ncRNAs including miRNAs and thus can influence the biological function of the target cells [63]. EV-encapsulated miRNAs can be used as predictive, diagnostic, and prognostic tools [64]. Among the several miRNAs, miR-21 and miR-126 have special attention because of their involvement in multiple diseases [54, 65, 66]. An aberrant expression of miR-21 has been reported in breast, gastric and lung cancer [67]. In RCC, several studies described that upregulation of miR-21 is associated with reduced survival [68], indicating a pathogenetic role of miR-21 as onco-miRNA. miRNA-126 is mapped within its host gene epidermal growth factor like-7 (EGFL-7) and is highly expressed in vascular endothelial cells [69]. By regulating VEGF pathway, miR-126 plays an important role in angiogenesis, lymph-angiogenesis and vessel integrity in endothelial cells as well as in cancer cells [70]. Moreover, miR-126 acts as a tumour suppressor and is downregulated in various cancer types including leukaemia, breast, gastric, prostate cancer. RCC and in NSCLC [71]. The miR-21 has been found aberrantly expressed in MPE, particularly in the context of NSCLC [47, 51]; this evidence supports the biological relevance of miR-21 in NSCLC-PE. The current study provides new insights into the role of miR-21-5p contained in sEVs isolated from MPE of NSCLC patients in cancer progression.

**Fig. 9:**
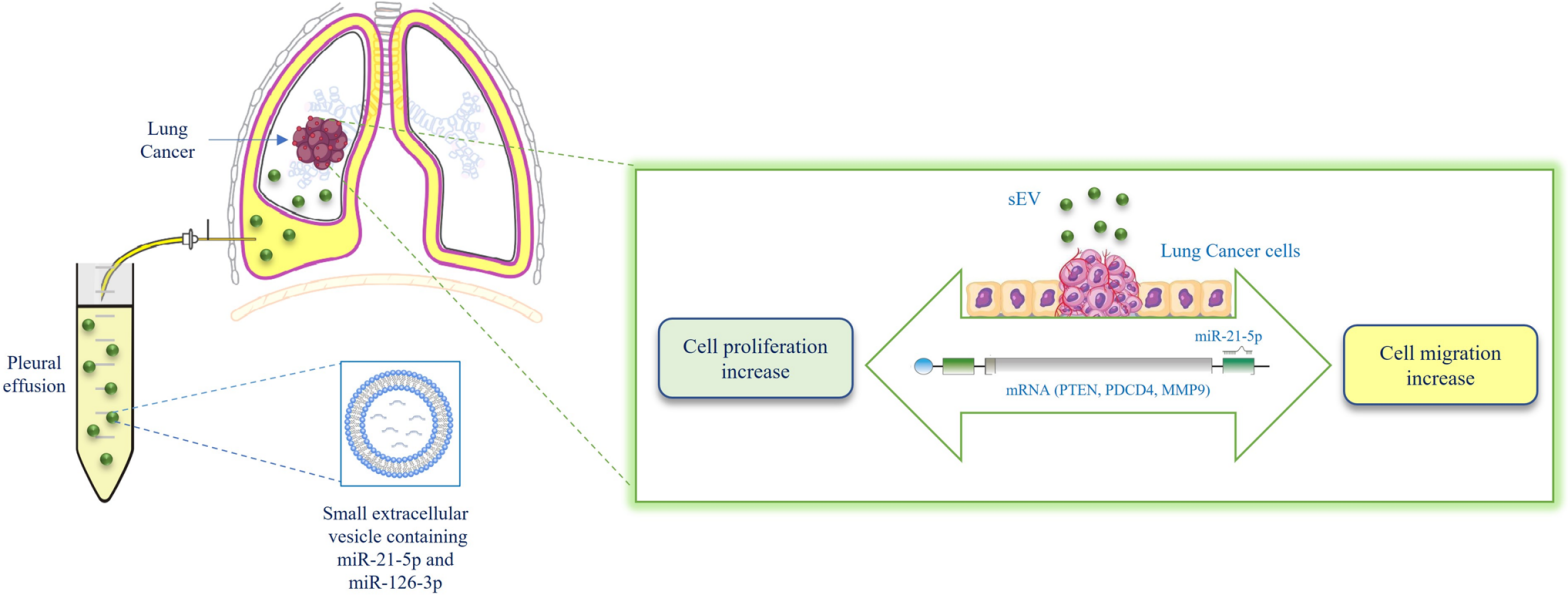
Proposed model of NSCLC-PE-sEV effects on LC progression: sEVs isolated by NSCLC-PE contains miR-21-5p that inhibits its target genes PTEN and PDCD4 on NSCLC cells, inducing cell proliferation and migration

PE is anatomically close to the lung and has important functions in the chest's milieu; it can be useful for monitoring NSCLC biomarkers [72]. EVs in PE originate from impaired lung tissue or cancer cells and it was demonstrated that they are involved in different steps of tumour progression such as proliferation, survival, pre-metastatic niche formation, angiogenesis, extracellular matrix degradation, stroma remodelling, immune escape, drug resistance [9, 73]. It was demonstrated that EV-miR-21 expression level in the pleural effusions is upregulated before pleural exposure of cancer cells [51]. EV-miR-21 upregulation at the pre-dissemination stage may promote cancer cell survival in the pleural cavity and it may create a premetastatic niche by inducing the mesothelial to mesenchymal transition (MMT) in the pleural cavity [51]. Our results indicate that NSCLC-PE-EV-miR-21 is responsible also for cancer cellproliferation and migration. The sEVs collected by NSCLC-PE induce the upregulation of miR-21-5p into NSCLC cells as confirmed by the gain and loss of function studies, performed using miR-21 mimic and inhibitor. Several target genes of miR-21 have been identified, including the onco-suppressors PTEN, PDCD4 [74]. An increase of miR-21 expression can upregulate MMP9 levels through indirect mechanisms, primarily by suppressing negative regulators of signalling pathways that control MMP9 transcription, inducing cancer progression [41]. It was demonstrated that miR-21-5p induces NSCLC cell proliferation and migration targeting PTEN, PDCD4 [37, 75]. These target genes of miR-21-5p are implicated in lung cancer progression and are involved in the upregulation of MMP-9, an endopeptidase that modulates the TME [76]. miR-21-5p also promotes NSCLC metastasis increasing the level of MMP9 [77]. PTEN has been considered an effective suppressor regulator of many cancers, including NSCLC, involved in the cell cycle, apoptosis, and tumour progression; it exerts a suppressive effect on tumours through the AKT pathway, by negatively regulating the intracellular levels of PI3K [78]. Our data, for the first time, indicate that COLO699 cells treated with NSCLC-PE-EVs decrease PDCD4 and PTEN gene expression, confirming the role of NSCLC-PE-EVs in LC cell proliferation and migration. Functionally, an increase of miR-21-5p that in turn induces the decrease of PTEN and consequently can increase AKT phosphorylation, induce cell proliferation and survival [75]. Moreover, PDCD4 is a well-known tumour suppressor gene in several cancers, including LC, and its post-transcriptional activity is directly controlled by miR21. PDCD4 affects the apoptotic machinery and inhibits the translation of several oncoproteins by suppressing the activity of eukaryotic initiation factors 4 A and 4G (eIF4A, eIF4G) and impacts gene transcription by interacting with JNK/c-Jun/AP-1 pathway [79]. Nuclear PDCD4 expression decreases during oncogenesis and can be considered a potential indicator of malignant transformation [80, 81]. Our results also indicate that sEVs collected by NSCLC-PE increase MMP-9 transcription into LC. MMP9 belongs to family of zinc-dependent endopeptidases with important functions in extracellular matrix remodelling during development, inflammation, and wound repair processes [82, 83]. As regulator of the tumour microenvironment, MMP9 has key roles in cancer initiation, development, and progression via multiple mechanisms. The link among mir-21-5p, PDCD4 and MMP-9 was already demonstrated: in HepG2 cells, the inhibition of miR-21 expression reduces cell migration and invasion by upregulating PDCD4 and downregulating downstream molecules such as MMP-9 involved in important signalling pathway [84]. Based on this evidence our results suggest for the first time that NSCLC-PE-sEVs-miR-21 can promote migration and invasion in NSCLC through the miR-21-PDCD4-MMP9 pathway. Overall, our data show that EVs collected from NSCLC-PE are involved in the intricate network of the pleural microenvironment inducing LC proliferation and migration, via miR-21-5p overexpression (Fig. 9). Further studies are needed to investigate the different cargo, such as proteins and ncRNAs, of NSCLC-PE-EVs with respect to CHF-PE-EVs, to clarify the molecular mechanisms underlying the different effects of EVs collected by NSCLC-PE and CHF-PE on cancer progression. This study demonstrates dysregulation of EV-miR-21-5p and EV-miR-126-3p in NSCLC-PE suggesting the potential of MPE use as a direct access for the analysis of these EV-miRNAs as biomarkers.

## Supplementary Information

Below is the link to the electronic supplementary material.

Figure 1S. COLO699 cells internalize CHF-PE-sEVs: Confocal microscopy analysis of COLO699 cells treated, for 1 and 3 hours, with CHF-PE-sEVs (20 μg/ml). COLO699 cells were stained with ActinGreen (green), nuclear counterstaining was performed using Hoescht (blue); CHF-PE-sEVs were labelled with PKH26 (red). Red channel (a and c), merge images (b and d). Detail of merge figure (e and f). Magnification (60x). Scale bar 50 µm. Supplementary file1 (JPG 586 KB)

Figure 2S. Semiquantitative analysis of sEVs internalization. The uptake of 20 μg/ml of NSCLC-PE-sEVs (Figure 2Sa) and CHF-PE-sEVs (Figure 2Sb) labelled with PKH-26 by COLO699 cells, at 1 and 3h. Semiquantitative analysis of red fluorescence performed with Image J software. Supplementary file2 (JPG 109 KB)

Figure 3S. Representative images of COLO699 cell migration. (a) COLO699 cells untreated, (b) treated with NSCLC-PE-sEVs (20 μg/ml), (c) NSCLC-PE, (d) NSCLC-PE-sEV-deprived, (e) CHF PE-sEVs (20 μg/ml), (f) CHF-PE, (g) CHF-PE-sEV-deprived. Scale bar 100 µm. Supplementary file3 (JPG 2007 KB)

## Data Availability

The data generated in the present study are included in the figures of this article.
