# Supplementary figures and images for: Effects of small extracellular vesicles isolated from pleural effusion on lung cancer cell proliferation and migration
Source: Hum Cell. 2025 Nov 15;39(1):10. doi: 10.1007/s13577-025-01322-8 (PMC12619733; doi:10.1007/s13577-025-01322-8)

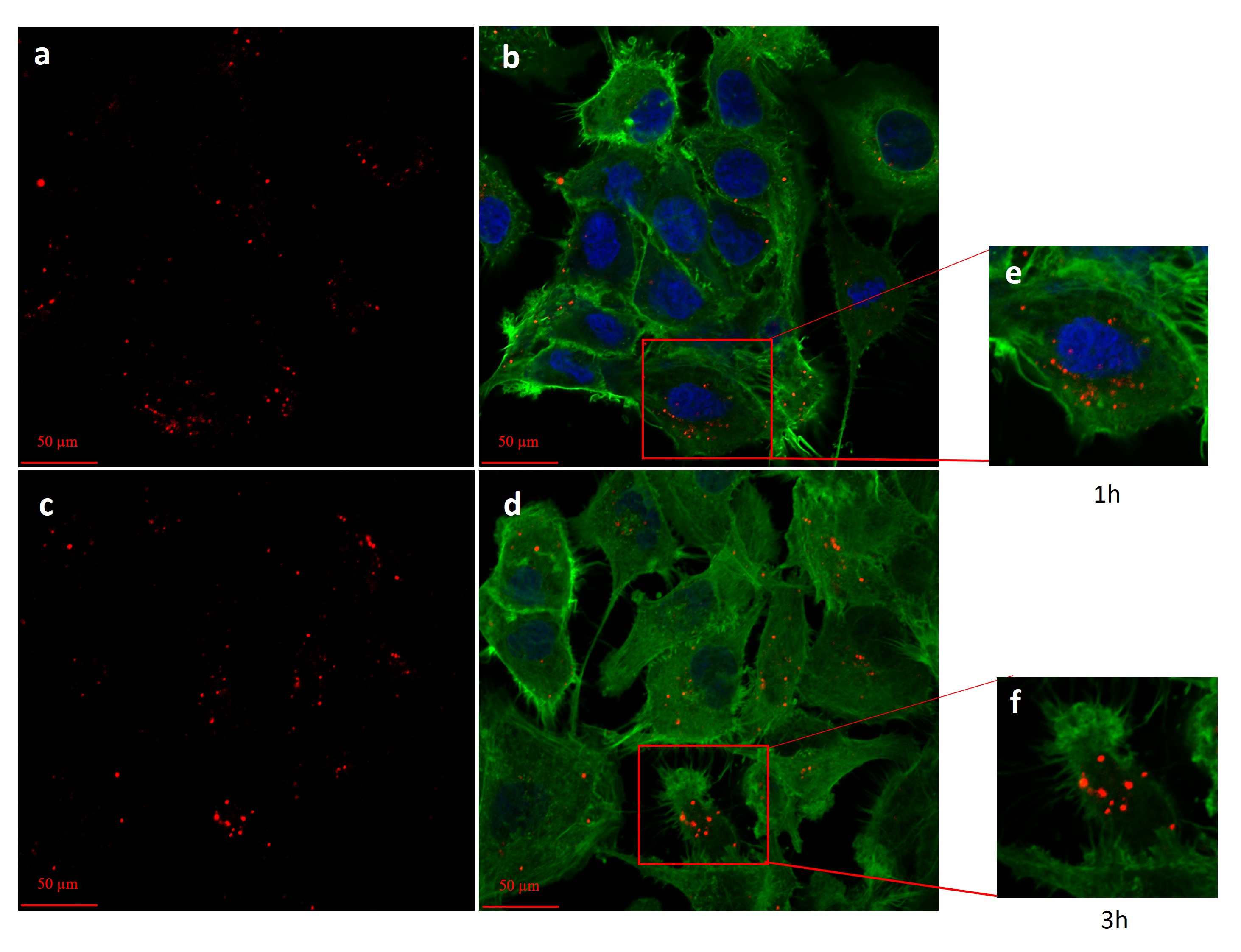

Supplement: Supplementary file 1 — Figure 1S. COLO699 cells internalize CHF-PE-sEVs: Confocal microscopy analysis of COLO699 cells treated, for 1 and 3 hours, with CHF-PE-sEVs (20 μg/ml). COLO699 cells were stained with ActinGreen (green), nuclear counterstaining was performed using Hoescht (blue); CHF-PE-sEVs were labelled with PKH26 (red). Red channel (a and c), merge images (b and d). Detail of merge figure (e and f). Magnification (60x). Scale bar 50 µm. Supplementary file1 (JPG 586 KB) [file 13577_2025_1322_MOESM1_ESM.jpg]

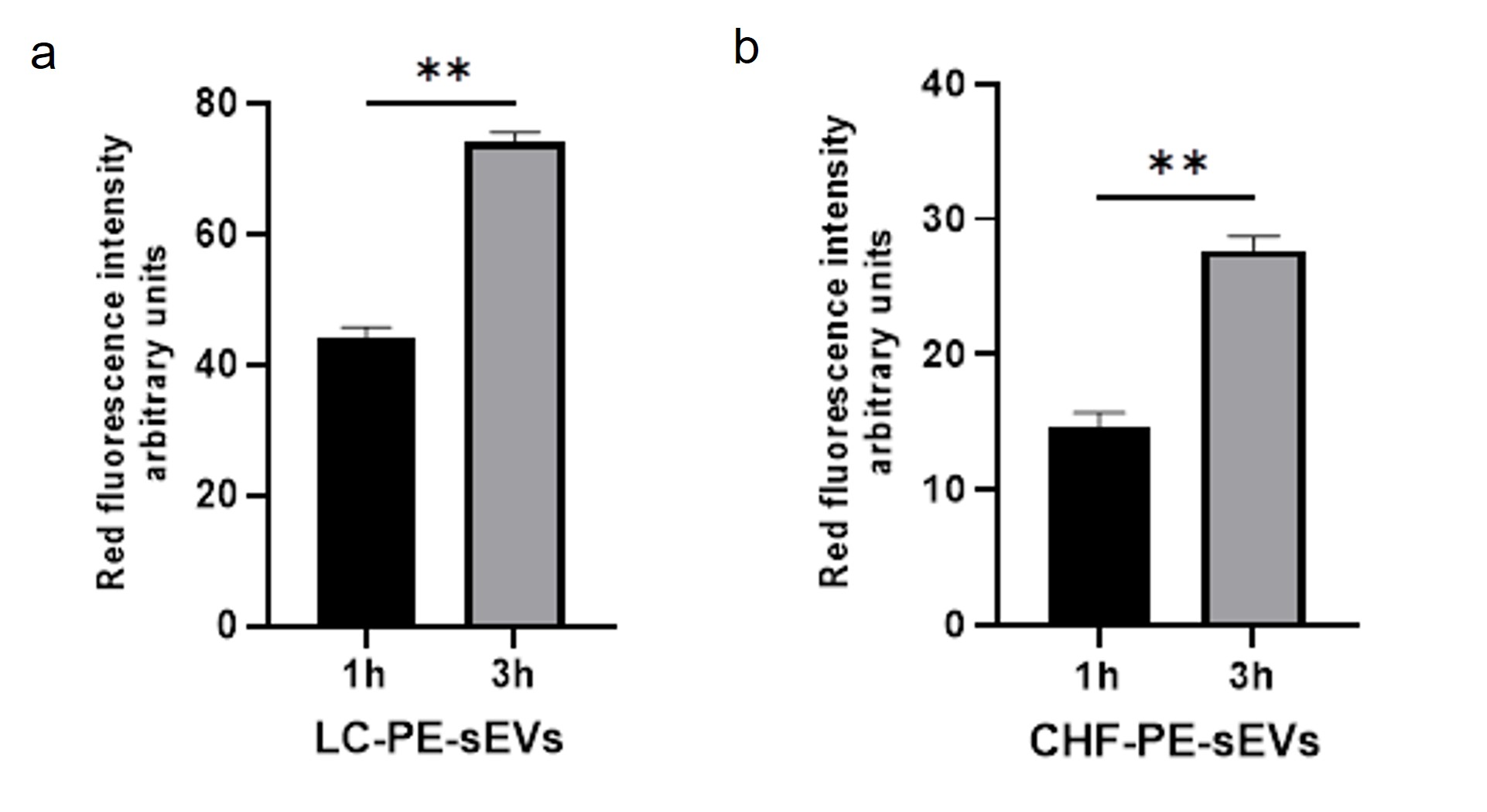

Supplement: Supplementary file 2 — Figure 2S. Semiquantitative analysis of sEVs internalization. The uptake of 20 μg/ml of NSCLC-PE-sEVs (Figure 2Sa) and CHF-PE-sEVs (Figure 2Sb) labelled with PKH-26 by COLO699 cells, at 1 and 3h. Semiquantitative analysis of red fluorescence performed with Image J software. Supplementary file2 (JPG 109 KB) [file 13577_2025_1322_MOESM2_ESM.jpg]

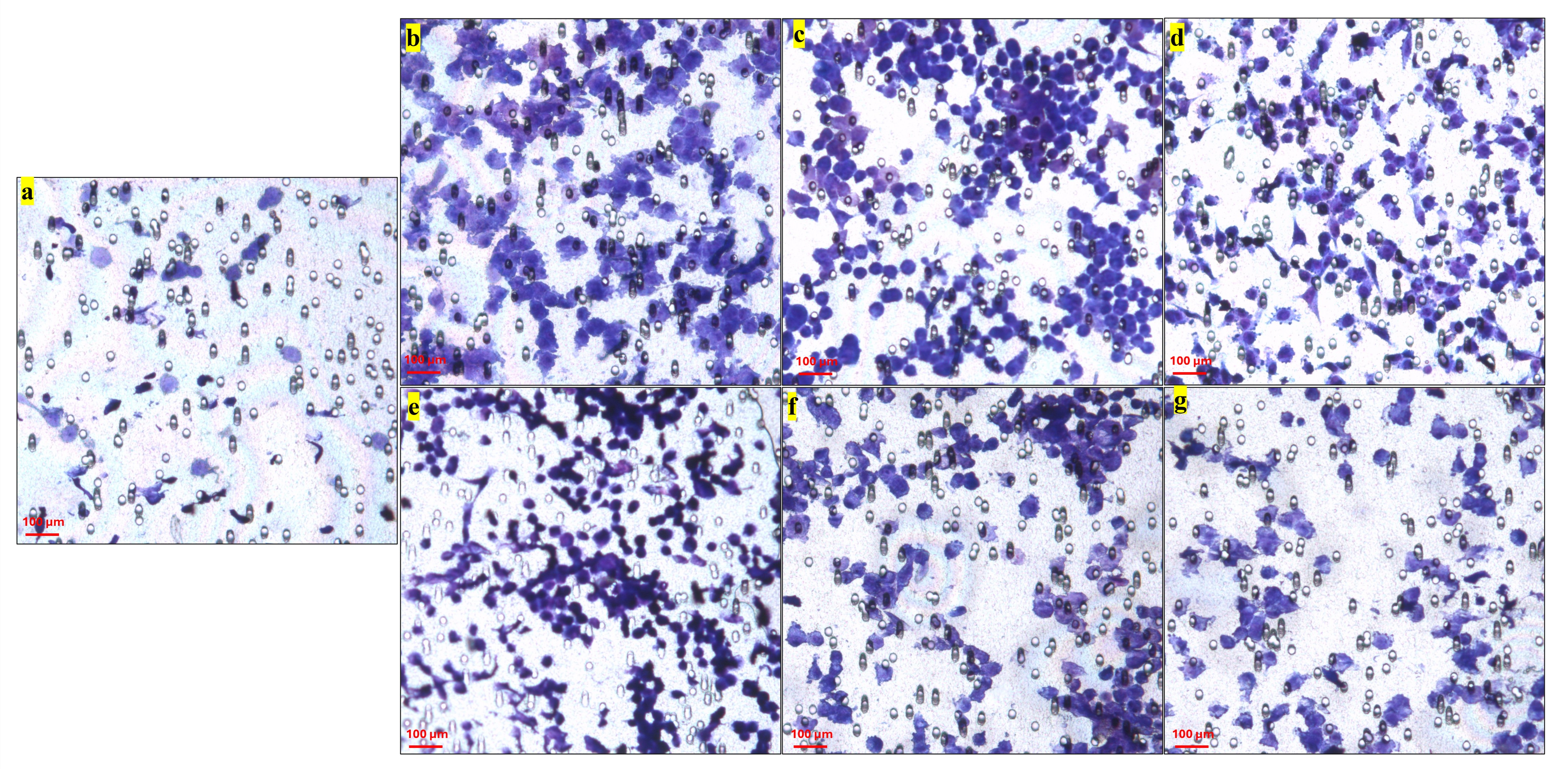

Supplement: Supplementary file 3 — Figure 3S. Representative images of COLO699 cell migration. (a) COLO699 cells untreated, (b) treated with NSCLC-PE-sEVs (20 μg/ml), (c) NSCLC-PE, (d) NSCLC-PE-sEV-deprived, (e) CHF PE-sEVs (20 μg/ml), (f) CHF-PE, (g) CHF-PE-sEV-deprived. Scale bar 100 µm. Supplementary file3 (JPG 2007 KB) [file 13577_2025_1322_MOESM3_ESM.jpg]
